# Effect evaluation of a tele-neurologic intervention in primary care in a rural area in Germany—the NeTKoH study protocol of a stepped-wedge cluster randomized trial

**DOI:** 10.1186/s12913-023-09724-w

**Published:** 2023-07-14

**Authors:** Kerstin Wainwright, Imke Mayer, Ana S. Oliveira Gonçalves, Ricarda S. Schulz, Simone Kiel, Jean-François Chenot, Agnes Flöel, Felix von Podewils, Anselm Angermaier, Tobias Kurth, the NeTKoH Consortium

**Affiliations:** 1 Institute of Public Health, Charité – Universitätsmedizin Berlin, Berlin, Germany; 2 Department of General Practice, Institute for Community Medicine, University Medicine Greifswald, Greifswald, Germany; 3 Department of Neurology, University Medicine Greifswald, Greifswald, Germany

**Keywords:** Tele-neurology, Teleconsultation, Telemedicine, Primary care, Outpatient general practitioner, Health care management, Integrated care, Stepped-wedge cluster randomized trial

## Abstract

**Background:**

Neurological disorders account for a large and increasing proportion of the global burden of disease. Therefore, it is important to strengthen the management of neurologic care, particularly in rural areas. The use of tele-neurology in primary care in rural areas is internationally considered to have the potential to increase access to health care services and improve the quality of care in these underserved areas. NeTKoH aims to address the existing knowledge gap regarding the effects of a tele-neurologic intervention in primary care under real-world conditions in a rural area in Germany.

**Methods:**

NeTKoH is a cluster-randomized controlled trial with a stepped-wedge design involving 33 outpatient general practitioner’s (GP) offices (clusters) in a rural area in Northeast Germany. During 11 predetermined steps, all clusters are randomized before they cross over into groups from the control to the intervention arm. The targeted sample size is 1,089 patients with neurologic symptoms that are continuously being recruited. In the intervention arm, tele-neurologic consultations will be provided via a face-to-face video conferencing system with a neurologic expert at a university hospital. The control arm will receive usual care. The primary outcome is the proportion of neurologic problems being solved at the GP’s office. Secondary outcomes will comprise hospital stays and days, time until neurologic specialist appointments and diagnostics, patients’ health status and quality of life, outpatient and inpatient referrals.

A concurrent observational study, together with a process, implementation, and health economic evaluation, will also be conducted.

**Discussion:**

Using a stepped-wedge cluster design in a real-life situation can help with logistic challenges and enhance the motivation of the participating GPs, as all, at some point, will be in the intervention phase. With the additional implementation evaluation pertaining to external validity, an observational study, and a health economic evaluation, NeTKoH will be able to provide an extensive evaluation for health policy decision-makers regarding the uptake into standard care.

**Trial registration:**

German Clinical Trials Register (DRKS00024492). Date registered: September 28, 2021. Date and protocol version: June 2023, version 1.

**Supplementary Information:**

The online version contains supplementary material available at 10.1186/s12913-023-09724-w.

## Introduction

### Background and rationale

Neurological disorders account for a large and increasing proportion of the global burden of disease [1–3]. In Europe and also in Germany, almost 60% of the general population is affected by a neurological disorder [4]. Many neurologic disorders increase with age [2]. With respect to a progressively aging society in Germany, an increase in neurological diseases is expected [4].

Therefore, it is important to strengthen the management of care to avoid a lack of healthcare services for the population, particularly in rural areas [5]. West Pomerania in the state of Mecklenburg-West Pomerania in Northeast Germany is an area where outpatient care is foremost based on primary care due to the comparatively low density of specialist care available in the region. The increased need for regional specialist care and, thus, neurological expertise confronts local outpatient general practitioners’ (GP) offices, as well as neurologic specialists, with major challenges.

The use of telemedicine or telehealth is widely considered to have the potential to increase access to healthcare services and improve the quality of care in rural and underserved areas, where care with specialized services close to home is not guaranteed [6, 7].

Telemedicine is defined as “the use of electronic and telecommunication technologies to provide healthcare at a distance” [8]. It can take place in real-time via a video conferencing system and has been used for chronic disease management in integrated care [9].

Over the past 25 years, telemedicine has also been applied in neurology (tele-neurology), including clinical neurosciences and acute neurological care [10–12]. In primary care, neurologic telemedicine programs have also been set up using face-to-face video conferencing or teleconsultations. They indicate an improvement in the quality of care in rural areas, patients’ and primary care physicians’ satisfaction, as well as being associated with cost-savings [13–15].

Unlike acute neurological care, the use of tele-neurology in a primary care setting in an underserved rural area in Germany has, to our knowledge, not yet been established and evaluated. This study aims to close this knowledge gap.

### Objectives

The objective of neurologic teleconsultations in GP practices (NeTKoH) is to improve the quality of health care services in an underserved area in Germany through the use of tele-neurology in primary care. Patients with neurological symptoms and diseases in a rural community can receive neurologic specialty care close to home in their familiar GP practice via a face-to-face video conferencing system between the local outpatient GP and a neurologist from a neurology department of a university hospital in the region.

In addition, GPs are offered continued education in aspects of neurology by the neurology department of the participating university hospital. The teleconsultations between GPs and remote neurologists, as well as the continued education, are intended to strengthen on-site care and facilitate the regional care of patients. Furthermore, this interdisciplinary, integrated care approach is aimed at improving in a cost-effective way the coordination of health care management.

With a stepped-wedge cluster randomized controlled trial under real-world conditions, we aim to evaluate the effectiveness of tele-neurologic consultations in primary care on the healthcare system level (proportion of neurologic problems being solved in the GPs` offices, hospital stays, and days), on patients (time until neurologic specialist appointments and diagnostics, health status, quality of life), and health care providers (referrals). For the evaluation of NeTKoH, a stepped-wedge cluster randomized controlled design was chosen to facilitate the roll-out of the intervention and prevent the disappointment of local GPs not randomized to the intervention, as this intervention follows a piloting phase in the region regarding the feasibility of tele-neurology in primary care [16].

In addition, a process, implementation, and health economic evaluation together with a concurrent observational study regarding the effects of continued neurologic education will also be undertaken in connection with this project. This protocol will focus on the trial part of NeTKoH.

## Methods/Design

The study protocol of NeTKoH adheres to the reporting guidelines defined by the SPIRIT 2013 [17] statement (see Supplemental file 1), the TIDieR checklist [18], CONSORT [19] and its extension for stepped-wedge cluster randomized trials (SW-CRT) [20].

### Design

NeTKoH is a cluster randomized controlled trial following a cross-sectional stepped-wedge design with continuous recruitment and a short exposure period*.*

During NeTKoH, the tele-neurologic intervention will be rolled out sequentially to 33 GP offices (clusters) from the control to the intervention arm. The control arm will receive care as usual. The overall trial period of 36 months and 17 days is divided into 11 "steps" with a length of 12 weeks each. Individual steps are extended by 1—2 weeks to account for school vacation periods.

**Fig. 1:**
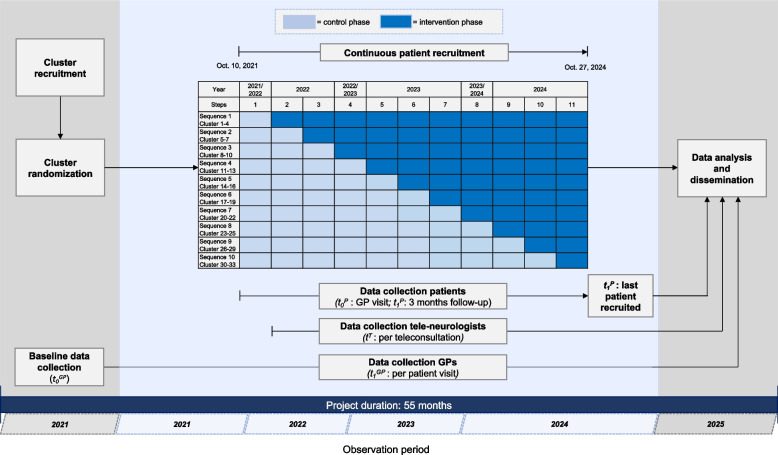
Schematic illustration of roll-out and timeline of the NeTKoH trial

In the 1st step of this pragmatic trial, all clusters start in the control phase. At each subsequent step, 3 to 4 randomly selected clusters will switch to the intervention phase. At the beginning of the 11th step, all clusters will have entered the intervention phase (see Fig. 1). This design was chosen under the given time limits by the funding agency to take the impact of COVID-19 on primary care settings in fall 2021 into account. The originally planned design consisted of 12 steps, with 3 randomly selected clusters switching at each step. A cluster was defined as a GP practice established according to the German health care planning requirements.

### Study setting, eligibility criteria, and recruitment

The study is conducted in an outpatient primary care setting in rural West Pomerania in the state of Mecklenburg West Pomerania in Northeast Germany. Eligible study sites had to be (1) located in the region of West Pomerania, (2) managed by GPs or internists, (3) who see patients with statutory health insurance, and (4) have the technical and spatial requirements for establishing teleconsultations.

GPs in the region were contacted by an informational letter about the project via the local Association of Statutory Health Insurance Physicians and the regional university hospital University Medicine Greifswald (UMG). Informational meetings regarding the intervention were held 10 months before the start of the trial. After expressing written consent, interested GPs were visited by the project planners of the NeTKoH group. The study design was explained, and further instructions, including a handout, were given. A cooperation agreement was then signed with 33 GPs. All participating GPs were granted a monetary incentive of 100 € per included patient to foster their cooperation. A list of study sites can be obtained from the NeTKoH study group.

### Participants, eligibility criteria, and recruitment

Patients admitted to NeTKoH have to be (1) at least 18 years old, (2) present themselves with neurological symptoms (any symptoms that can warrant a referral to a specialist in neurology) to the participating GP, (3) members of a statutory health insurance, and (4) able to give informed consent.

Due to the variance and broadness of neurological symptoms, the review of an extensive symptom list by the GP during the patient appointments was deemed not realistic by experts involved in the project. In addition, symptom categories always represent a simplification that does not do justice to the complexity of neurological diseases. Moreover, the project does not focus on a specific neurological disease. Therefore, due to medical, pragmatic, and methodological reasons, the "presence of a neurological deficit according to the assessment of the GP" or the question of the GP "Would you like to have a neurological teleconsultation?" was considered as a more appropriate way to screen potential participants.

Patients are excluded if the GP considers them to have a neurological emergency (e.g., stroke) that requires prompt inpatient attention or if they are unable to give informed consent. Patients can only be admitted once with the same neurological problem to the trial.

Given the open cohort design, patients are continuously screened and recruited by the GPs when they present with a neurologic symptom.

### Intervention

During the intervention phase of the trial, all clusters will receive neurologic teleconsulting. In order to do so, they will be equipped with a certified telemedical system (VIMED® Praxis) provided by the company MEYTEC (Werneuchen, Germany). This telemedicine device is a face-to-face video-conferencing tool connected with the department of neurology at UMG. It allows for a remote audiovisual interaction between a tele-neurologist located at UMG and the participating GP, together with the patient. The NeTKoH video-conferencing system is a hardware solution with integrated microphones and speakers, as well as a remotely controllable camera. Internet access is achieved via mobile radio.

Eligible patients can be included in the study by the participating GPs after informed consent is given by the patients in the course of their consultation visit. Patients, who are willing to participate in the study, will be asked to wait while a medical assistant of the GP’s office activates the telemedical system in a separate room in order to start the registration process for the consultation electronically. Through the registration, a medical assistant located at the neurology department (UMG), is notified and schedules an appointment. Thereby, a notification appears on the telemedicine device located in the GPs office. The GP’s office will then schedule the consultation and will inform the patient about the planned start of the consultation with the tele-neurologist, which then takes place in the GP’s examination room. The teleconsultation, which is supposed to be limited to a maximum of 15 min, includes questions about symptoms, diagnostics performed, treatments, referrals, medications, and a brief symptom-oriented clinical examination. After the consultation, the findings and recommendations are electronically documented and a print-out in the GP’s office is created.

During the time a cluster is randomized to the control phase, treatment will be provided according to ‘usual care’ after informed consent is given by the patient. This means that clusters delivering the control condition will not be equipped with a telemedicine device, and patients will have no access to a tele-neurologist during their GP visit. Treatment will follow local standards.

### Outcomes

Outcomes pertain to the health care system (macro level), the institution (meso level) as well as the individual (micro level).

The primary outcome is the proportion of neurologic problems being solved in the GPs’ practices, measured by the number of patients without an outpatient referral (neurologic/other specialists), inpatient referrals (hospital), or other.

The secondary outcomes include on the macro level: (1) number of hospital stays, (2) length of hospital stay, on the meso level (3) number and types of referrals, and on the micro level (4) time until neurologic appointments, (5) time until specialty diagnostics, (6) quality of life, and (7) health status.

### Participant timeline

The data for the primary outcome will be measured after the GP appointment (*t*_*1*_^*GP*^) with a self-complete paper survey (control arm) and collected in the NeTKoH database by the tele-neurologist (intervention arm) at *t*^*T*^. At baseline (*t*_*0*_^*GP*^), organizational and demographic data of the settings and GPs will be collected with a survey by the study center.

Secondary outcome data (see Table 1) regarding the enrolled patient’s quality of life and health status will be assessed for both groups after the GP appointment regarding a neurologic problem (*t*_*0*_^*P*^) with the self-complete EQ-5D-5L. In order to minimize interference in the GP offices’ everyday routine, only this time point (*t*_*0*_^*P*^) was deemed feasible. Patients’ demographics will also be collected in a self-complete paper survey. Their disease characteristics will be documented by the GPs at *t*_*1*_^*GP*^ in the GP survey. The subsequent treatment (referrals) of patients and further process-related data will be documented by the tele-neurologist in the NeTKoH-database at *t*^*T*^. Just-in-time care endpoints, such as time until a neurologist appointment, and specialty diagnostics, as well as the number of hospital stays, will be assessed at *t*_*1*_^*P*^ for both groups taken in the form of a standardized telephone interview 3 months after the patients’ initial GP appointment. All patient answers to the standardized questions at the 3-month follow-up (*t*_*1*_^*P*^) will be electronically documented by the study team. The patients’ length of stay (LOS) at the hospital between *t*_*0*_^*P*^ and *t*_*1*_^*P*^ will be collected via claims data provided by the two participating health insurance providers.

**Table 1:** NeTKoH—secondary outcomes, timepoints^a^, and instruments

Outcome	Timepoint	Measurement instrument
Subsequent treatment (referrals): GP/neurologist/other medical specialist/inpatient care/others *(intervention group – control group)*	*t*_*1*_^*GP*^*, t*^*T*^	Questionnaires GPs, NeTKoH-database
Time until appointment neurologist (days) *(intervention group – control group)*	*t*^*T*^ *t*_*1*_^*P*^	*Intervention group*: NeTKoH-database *Control group*: standardized phone interview
Time until appointment for indicated initial specialty diagnostics: a) cMRT, cCT b) electrophysiology of nerves/muscles, c) ultrasound of brain supplying vessels, d) Electroencephalography *(intervention group – control group)*	*t*_*1*_^*P*^	Standardized phone interview
Number of hospital stays (for *t*_*0*_^*P*^*- t*_*1*_^*P*^) *(intervention group – control group)*	*t*_*1*_^*P*^	Standardized phone interview
Number of hospital days/length of stay (for *t*_*0*_^*P*^*- t*_*1*_^*P*^) *(intervention group – control group)*	*t*_*1*_^*P*^	Claims data
Quality of life *(intervention group – control group)*	*t*_*0*_^*P*^*, t*_*1*_^*P*^	EQ-5D-5L (hybrid): self-complete paper (*t*_*0*_^*P*^*)* and standardized phone interview (*t*_*1*_^*P*^)
Health status *(intervention group – control group)*	*t*_*0*_^*P*^_*,*_* t*_*1*_^*P*^	EQ-5D-5L VAS (hybrid): self-complete paper (*t*_*0*_^*P*^*)* and standardized phone interview (*t*_*1*_^*P*^)

*GP* general practitioner, *t*^*T*^ after teleconsultation,* t*_*0*_^*P*^ directly after initial GP appointment (patients), *t*_*1*_^*P*^ after a 3-months follow up (individual for patients), *t*_*1*_^*GP*^ directly after initial GP appointment (GPs), *cMRT* cranial computed magnetic resonance imaging, *cCT* cranial computed tomography, ^a^timepoints assessed, *VAS* visual analog scale

### Data collection, management, and quality control

Paper surveys for patients and GPs are used to increase the cooperation of GPs and patients during the trial, according to local experts in the study group. However, all paper surveys were created using the software program evasys [21], so that an automatic export of the survey answers at the study center of the participating university hospital (UMG) will be possible for the subsequent evaluation at Charité – Universitätsmedizin Berlin (Charité). The patient and GP paper surveys will be picked up once a month from the GP offices by UMG study personnel.

The standardized telephone interview at 3-month follow-up for patients will be based on a survey, pre-programmed in an evasys web surface, which will be filled out by the interviewer during the call. Since we are expecting attrition to occur, especially at the 3-months follow-up, reasons for dropouts and loss-to-follow-up will be collected.

The NeTKoH-database was programmed using automatic plausibility checks and mandatory fields for data entry. Technical training of the GP offices regarding the use of the teleconsulting system will take place prior to the start of the intervention. On-site monitoring, a feedback loop, and measures to inquire regarding implausible or missing data by the study personnel will be instituted. The study personnel will receive training for the documentation in the NeTKoH database and the evasys web surface. All surveys were piloted before the start of the intervention.

All data collection, handling, transferring, processing and storage will follow the project’s comprehensive data protection concept approved by the responsible data protection officers. The primary and secondary data of NeTKoH will be securely transferred from the data integration center at UMG to the independent evaluation team at Charité for the final analysis and provision of findings to the funding agency. Trained researchers of the evaluation team will code, enter and store the data on the secure Charité server. Only the evaluation team will have access to the final data set.

### Statistical methods: sample size and power calculations.

The study includes 33 clusters in the region of West Pomerania, in which the participating university hospital is located. Sample size calculations were based on the Hussey & Hughes approach [22] for stepped-wedge cluster design for analysis with fixed time effects and random cluster effects.

The sample size calculation was based on the primary endpoint. As a basis for this calculation, we took the assumption that in the control group, the proportion of outpatient neurological symptoms that can be solved in the GP's practice (i.e., there is no subsequent neurological or special outpatient consultation or inpatient referral) is 0.2 (20%). We expect a 50% improvement (i.e., a 50% increase in effect size on the risk difference scale) in the intervention group, corresponding to an expected proportion of 0.3 (30%) in the intervention group.

With a total sample size of 1,089 participants (corresponding to 555 patients included in the control phase and 534 patients included in the intervention phase) at a type I error rate of 0.05, and an assumed coefficient of variation of 0.25, the study has a power of 80%, at a coefficient of variation of 0.20 and a power of 81% to measure the assumed effect size or a larger effect. Due to the nature of the intervention and the immediate collection of the primary endpoint data following the intervention, no attrition pertaining to this endpoint can be expected.

To achieve this number of cases, the stepped-wedge cluster randomized trial (CRT) must recruit, on average, at least 3 patients meeting the inclusion criteria, per cluster and step, to perform an appropriate analysis for this design.

To examine secondary endpoints (except those using claims data), all patients included during the study can be analyzed. Based on an estimate from experts of the NeTKoH study group, we assume an average demand of 6 to 8 neurological consultations per GP per month, which means about 3,168 neurological consultations per year in the whole region. Of these, about 25% of patients* (N = 792 per year, N = 66 per month) are insured by the participating statutory health insurances AOK Nordost and Techniker Krankenkasse. Assuming a loss of 30% due to non-participation or loss-to-follow-up, complete data of about 554 neurological consultations of the AOK Nordost and Techniker Krankenkasse patients per year and of about 1,269 to 1,690 patients during the whole study period are possible.

For the secondary endpoints, which are patient-reported outcomes, an expected dropout of 30% was included in the calculations. Using a t-test with a two-sided type I error of 0.05, a mean difference of 0.20 (Cohen's d, small effect), and a case number of 841 patients (310 in the intervention group and 531 in the control group), a power of 0.80 was obtained. This calculation was performed using the software G*Power 3.1.9.7 [23].

### Sequence generation and intervention allocation

Following the considerations of a stepped-wedge design, GP practices were randomized to switch from the control into the intervention arm according to the pre-assigned dates of such a switch (see Fig. 1). The sequences to cross over from the control to the intervention arm were determined with a computerized randomization generator prior to the start of the trial by an independent investigator of the evaluation team. Except for the 1st, second to last, and last sequence in which 4 clusters will switch, each sequence is assigned to 3 clusters. All randomized clusters will be informed 4—6 weeks prior to their switch in order to prepare for the roll-out of the intervention. The entire randomization schedule of the clusters will not be shared with the participating clusters.

However, concealment of the sites and blinding of the GPs is not possible by the nature of the trial. Patients will only be aware of the allocation sequence after having given informed consent and once the GP discloses it to them. Then they will be aware of whether they are in the intervention or control group. Although data analysts will also not be blinded, we do not anticipate major bias to influence the analyzed effects.

### Statistical analysis

The principal analysis focuses on the effect of the tele-neurological intervention, defined by the primary outcome, namely the proportion of neurologic problems being solved in the GPs’ practices, to answer the following question: *Do neurological teleconsultations in GP practices lead to an increase in the proportion of patients with neurological issues resolved in the GP practice in comparison to care as usual?*

A generalized linear mixed effects model will be used, in accordance with the sample size and power considerations above, to adjust for time-specific confounding and intra-cluster correlations. We assume time and intervention effects are common to all clusters, and model them as fixed effects. The random cluster effects allow accounting for intra-cluster correlation. Related sensitivity analyses will be described in the Statistical Analysis Plan.

We will report point estimates of the intervention effect along with 95% confidence intervals on absolute and relative scales (respectively measured as rate difference and rate ratio). Estimated correlations and time coefficients will also be reported.

The secondary analyses will assess the secondary outcomes and consist of testing the different study assumptions also using generalized linear mixed effects models (for secondary outcomes 1, 2, 4, 5, 6, 7), as well as a cluster-adjusted chi-squared test (for secondary outcome 3). For all analyses, we consider a significance level of 0.05.

The statistical analyses will be performed using R v4.2.2 (or higher, [24]). A comprehensive, detailed Statistical Analysis Plan, with details on handling missing values and ancillary analyses at individual and cluster levels, will be prepared before data locking and extraction for the statistical analysis.

### Observational study

A concurrent prospective observational cohort study aimed at the individual GP level is planned alongside the trial to evaluate the need for continued neurological education. In this observational study, the effectiveness of neurologic continued education courses offered at regular intervals to GPs by neurologists will be assessed. During the NeTKoH study, these optional sessions are offered once every quarter of the year for continued education credits to the participating NeTKoH-GPs. The analysis of the cohort study will include GPs who participate in at least one neurological training during the entire NeTKoH study.

The primary outcome of this observational study will be the change in self-reported needs in neurological continued education between baseline (i.e., at the study initiation) and end of the study period (after recruitment of the last patient into the trial).

### Process and implementation evaluation

Using the Reach—Efficacy/Effectiveness—Adoption—Implementation—Maintenance (RE-AIM) framework [25], the implementation of NeTKoH will be evaluated to ensure that both interventions (teleconsulting, neurologic continued education) are delivered and implemented as planned and to provide insights on their generalizability. For instance, for the RE-AIM dimension “Implementation,” key elements of the tele-neurologic intervention, such as the number of requested and established teleconsultations, their duration, but also adverse events, e.g., technical problems will be measured. Moreover, the satisfaction of patients and GPs with teleconsultation, as well as the planned adoption of the tele-neurologist’s recommendation, will be analyzed using descriptive statistics. The program costs pertaining to the German healthcare context will also be calculated.

### Health economic evaluation

The economic evaluation will be carried out alongside the trial. It will consist of a cost-utility analysis and three cost-effectiveness analyses. The outcome considered for the cost-utility analysis will be quality-adjusted life years (QALYs) derived from the EQ-5D-5L using German population weights [26]. Effectiveness measures will include the proportion of neurologic problems being solved at the GP’s office, the number of hospital stays, and the length of stay at the hospital. We will include all relevant additional trial costs of the intervention compared to standard care, as well as patient-specific costs stemming from claims data of the participating health insurances. The economic evaluation will be performed from a statutory health insurance perspective.

## Discussion

The increase in neurologic disorders requires strengthening of management and access to neurologic care, particularly in resource-poor rural areas. Tele-neurology in the form of teleconsultations between outpatient GPs and neurologic experts in underserved areas has been identified as an appropriate measure to counter this development [27].

For such interventions in primary care, a CRT with settings as the unit of randomization and patients nested within those settings is particularly suited as it reduces the risk of contamination [28]. Conducting a stepped-wedge design, as an alternative design to a CRT [29] for a tele-neurologic intervention under real-world conditions in a rural area, also has pragmatic considerations. As all clusters receive the intervention by the end of the trial, enhanced recruitment and motivation are likely. Due to the sequential versus simultaneous roll-out of the intervention, this design also has logistic benefits for implementation in a rural area where participating settings can be distant from one another.

We expect that results from this trial will deliver insightful evidence on the effects of tele-neurologic consultations in primary care not only on the patient and organizational (provider) level but also with respect to the healthcare system.

There are several limitations to our study. This multi-center stepped-wedge design involving GP practices in the German public health care system can be vulnerable to secular trends, e.g., the COVID-19 pandemic and its impact on care. As with any trial in a real-world setting, we also expect a certain amount of missing information. However, these issues will be addressed with our analysis plan to account for such changes. Given the fact that the trial has to be integrated into the everyday care of the participating GP practices, only a finite number of parameters can be sampled to ensure uninterrupted workflow. However, our interdisciplinary approach during the evaluation planning phase, according to the Framework for Program Evaluation [30], involved clinicians (neurologists and GPs), biostatisticians, epidemiologists, health economists, and implementation specialists, which assured the necessary breadth of information to be collected. Due to local data protection regulations, no data on approached non-participant patients will be collected, thus limiting information on the representativeness of the reached population. This will be accounted for by providing detailed characteristics of the intervention participants.

The strength of NeTKoH is its pragmatic design, allowing for real-world evidence for a teleconsulting intervention in primary care accompanied by continued education for the participating GPs. Of note is that there will be no risk to the recruited patients and the trial will be monitored, and adverse issues will be reported.

Incorporating an evaluation of the implementation based on one of the most used approaches [31] will provide considerable information for policymakers to guide future use and possible scale-up of NeTKoH. Conducting a cost-effectiveness analysis will further provide key information for stakeholders.

### Trial status

The trial started on October 10, 2021. The last patient will be recruited on October 27, 2024. Results are expected in July 2025.

## Supplementary Information

**Additional file 1.** 

**Additional file 2.** 

## Data Availability

Not applicable.

## Abbreviations

CONSORT: Consolidated Standards of Reporting Trials
CRT: Cluster Randomized Trial
G-BA: Federal Joint Committee
GDPR: EU General Data Protection Regulation
GP: General Practitioner
LOS: Length of Stay
NeTKoH: Neurologic Tele-Consultations in GP Practices
QALY: Quality-Adjusted Life Year
RE-AIM: Reach—Efficacy/Effectiveness—Adoption—Implementation – Maintenance
SAP: Statistical Analysis Plan
TIDieR: Template for intervention description and replication
UMG: University Medicine Greifswald
VAS: Visual Analogue Scale
