# Supplementary material for: Effect evaluation of a tele-neurologic intervention in primary care in a rural area in Germany—the NeTKoH study protocol of a stepped-wedge cluster randomized trial
Source: BMC Health Serv Res. 2023 Jul 14;23:756. doi: 10.1186/s12913-023-09724-w (PMC10347790; doi:10.1186/s12913-023-09724-w)
Supplement: Supplementary file 2 — Additional file 2. [file 12913_2023_9724_MOESM2_ESM.docx]

**Supplemental File 2**

**The NeTKoH Consortium:**

Paula J. Filser^1^, Tobias Kurth^1^, Imke Mayer^1^, Ana S. Oliveira Gonçalves^1^, Ricarda S. Schulz^1^, Kerstin Wainwright^1^

Jean-Francois Chenot^2^, Simone Kiel^2^, Elisa Michalowsky^2^

Aiham Alkhayer^3^, Anselm Angermaier^3♣^, Agnes Flöel^3^, Verena Horn^3^, Wieland Köhn^3^, Malgorzata Kotarz-Boettcher^3^, Anne Krüger^3^, Felix von Podewils^3^, Cordula Weil^3^, Carl Witt^3^

Michael Böttcher^4^

Diana Graja^5^, Katrin C. Reber^5^, Olga Resch^5^

Juliane Rothe^6^, Jacqueline Syring^6^

**Affiliations**

^1^ Institute of Public Health, Charité – Universitätsmedizin Berlin, Berlin, Germany

^2^ Department of General Practice, Institute for Community Medicine, University Medicine Greifswald, Greifswald, Germany

^3^ Department of Neurology, University Medicine Greifswald, Greifswald, Germany

^4^ MEYTEC, Werneuchen, Germany

^5^ AOK Nordost, Potsdam, Germany

^6^ Techniker Krankenkasse, Hamburg, Germany

^♣^ Nominated consortium representative
